# Role of Newborn Hearing Screening Done Over One Year in a Tertiary Care Hospital: A Cross-Sectional Study

**DOI:** 10.7759/cureus.69521

**Published:** 2024-09-16

**Authors:** Anand Kumar G., Santosh Kumar Kamalakannan, Asha A., Harish Sudarsanan, Kumutha J.

**Affiliations:** 1 Pediatrics and Neonatology, Saveetha Medical College and Hospitals, Saveetha Institute of Medical and Technical Sciences (SIMATS) Saveetha University, Chennai, IND

**Keywords:** automated auditory brainstem response, bera, brainstem evoked response audiometry, hearing loss, newborn, newborn hearing screening, oae, otoacoustic emissions

## Abstract

Background: Newborn hearing screening (NHS) is universally acknowledged as a critical early intervention to prevent adverse developmental outcomes caused by undetected hearing loss. Despite its proven benefits, the implementation of NHS varies, especially in tertiary care hospitals that manage high-risk neonates. This study investigates the effectiveness and implementation of NHS protocols in these settings.

Methodology: This cross-sectional study was conducted in the Department of Neonatology at Saveetha Medical College, Chennai. All newborns delivered between January 2023 and December 2023 were included. Screening involved initial otoacoustic emissions (OAEs) tests, automated auditory brainstem response (AABR) followed by brainstem evoked response audiometry (BERA) for those who failed. Data on demographic characteristics, screening results, and follow-up compliance were collected and analyzed.

Results: A total of 1,398 neonates were screened. Initial screening resulted in 416 (29.7%) referrals. Follow-up screenings showed high compliance rates, with significant detections of hearing impairments through BERA. The screening was completed for 1,341 babies. Fifty-five babies were lost to follow-up. Of these, 2 babies (0.1%) with a high risk for hearing loss were diagnosed with bilateral severe hearing loss. The study also noted demographic factors such as kinship and obstetric history that might influence hearing loss risks.

Conclusions: NHS plays a vital role in the early detection and management of hearing impairments, which is crucial for preventing negative impacts on a child's development. This study advocates for the systematic implementation of NHS protocols in all tertiary care hospitals, especially those serving high-risk neonates, to ensure optimal developmental outcomes.

## Introduction

Newborn hearing screening (NHS) has been recognized globally as a critical early intervention measure. Its primary objective is the early identification of hearing loss, which, if undetected, can severely impact the linguistic, social, and cognitive development of children [1]. The importance of NHS lies in its potential to detect hearing impairments before they can affect these critical areas of development, thus facilitating timely medical or surgical interventions that can significantly improve the quality of life and developmental outcomes for affected infants [2].

Despite the proven benefits of universal neonatal hearing screening, its implementation varies significantly across different healthcare settings, particularly in tertiary care hospitals where high-risk neonates are often treated. These facilities, equipped with advanced medical technologies and specialized personnel, are ideal for the deployment of systematic NHS programs [3].

Otoacoustic emission (OAE) tests are quick, non-invasive, and can detect cochlear function by measuring sound waves produced in the inner ear in response to auditory stimuli. OAE is highly effective as an initial screening tool due to its simplicity and speed, allowing for widespread screening in newborns. However, they are less effective at identifying auditory neuropathy or neural hearing loss because they only assess the cochlea's response and not the auditory nerve or brain pathways [4].

Brainstem-evoked response audiometry (BERA) is a more comprehensive test that measures the brain's activity in response to sound. It can detect both cochlear and neural dysfunctions, making it an excellent diagnostic tool for confirming hearing loss identified through initial OAE screening. BERA is particularly useful in detecting auditory neuropathy and other types of neural hearing impairments, which OAE might miss. However, BERA is more time-consuming and requires a more controlled environment, which can be challenging with newborns [5].

Many healthcare settings use both OAEs and BERA in a two-step screening process to maximize the detection of potential hearing impairments. Initially, all newborns undergo OAE screening; those who refer or fail proceed to the more detailed BERA test [6]. This combined approach ensures a high detection rate while maintaining efficiency and cost-effectiveness. The use of both tests in tandem accommodates the strengths and limitations of each method, providing a comprehensive assessment of a newborn's auditory health [7].

Cross-sectional studies, such as the one proposed, are essential for assessing the current state of NHS practices within these hospitals. They provide valuable data on the prevalence of hearing loss, the effectiveness of screening protocols, and potential areas for improvement in screening procedures. For example, research conducted in a tertiary care hospital in Mexico City revealed a prevalence of hearing loss of 2.2 per 1000 newborns, emphasizing the importance of early diagnosis and habilitation [8]. Similarly, a study in Ethiopia highlighted significant factors associated with referral results in NHS, indicating specific areas where screening protocols could be optimized to reduce false positives and improve diagnostic accuracy [9].

A retrospective study by Lin et al. [10] found that using a two-step screening protocol, which includes both automated auditory brainstem response and OAEs, was more effective in detecting hearing loss in both general and high-risk infants.

This proposed research will fill a critical gap by providing up-to-date information on the profile of NHS in a tertiary care setting, thereby informing policy and operational guidelines that can enhance the effectiveness of hearing screening programs.

## Materials and methods

The cross-sectional study was carried out in the Department of Neonatology at Saveetha Medical College, Chennai, following approval from the Institutional Ethical Committee. All newborns delivered in the hospital from January 2023 to December 2023 were included in the study. High-risk neonates were identified based on the JCIH 2019 statement, and parental consent was obtained.

Inclusion criteria

The study included inborn and outborn babies who were admitted for either maternal or neonatal conditions.

Exclusion criteria

Newborns with severe multiple anomalies that were incompatible with life.

Routine hearing screenings were performed on all neonates. For babies admitted to NICU, the initial OAE screening took place before discharge or when the baby was transferred to the post-natal ward after 72 hrs of life. For babies who were directly transferred to the postnatal ward, OAE was conducted after 72 hours of life in both low-risk and high-risk newborns. A qualified audiologist conducted the OAE screening test in a soundproof room, with the presence of either the mother or caretaker. The infants were tested bilaterally while asleep, without sedation, in a quiet environment. The outcomes of the OAE tests were classified as either *pass* or *refer*.

Neonates who did not pass the initial screening underwent a second OAE screening within two weeks of discharge, after ensuring that the ear canal was clear of debris. Those who had been referred in the second screening underwent BERA. Proper probe sizes and insertion techniques were used, and an otoscopic examination of the external auditory canal and the tympanic membrane was performed using a Heine 3000 series otoscope.

A total of 1,398 high-risk neonates met the inclusion and exclusion criteria and were enrolled in the study after obtaining parental consent. 

The OAE screenings were performed using the Autonova Scan Transient Evoked Otoacoustic Emissions (TEOAE) device, which operates within a frequency range of 1.5 to 8 kHz. The machine is lightweight, with a timing window of 12.5 ms, and clicks delivered at a rate of 240 per second, consisting of standard transient clicks at 55 to 68 dB SPL (sound pressure level).

BERA was conducted using the IHS DUET system at a rate of 25/sec, an intensity ranging from 90 dB HL down to 30 dB HL, and a filter range of 30-3000 Hz. Latencies of waves I, III, and V were measured, and the diagnosed hearing deficits were classified as mild (21-40 dB HL), moderate (41-70 dB), severe (71-90 dB HL), and profound (≥90 dB HL). Neonates diagnosed with auditory disorders were advised to seek early hearing aid amplification and were referred to a rehabilitation center for appropriate management.

## Results

Table 1 shows the demographic details of the mother where the average age of the mothers was 26.33 years, with a standard deviation of 3.86 years. Consanguinity was reported in 9.9% of cases. In terms of obstetric history, a significant proportion of the mothers were multiparous, accounting for 64.4%, while primiparous mothers made up 35.6%. Pregnancy-induced hypertension (PIH) was present in 15.1% of the mothers, gestational diabetes mellitus (GDM) was observed in 18.3%, and thyroid problems were noted in 13.5%. Regarding prenatal findings, 8.4% of ultrasounds revealed abnormalities and premature rupture of membranes (PROM) occurred in 9.6% of the pregnancies. The amniotic fluid was reported as normal in 88.6% of the cases. When examining the mode of delivery, lower segment cesarean section (LSCS) births were more common, constituting 51.9% of the deliveries, while vaginal and assisted vaginal deliveries accounted for 42.9% and 5.2%, respectively (Table 2).

**Table 1:** Demographic characteristics of a mother. LSCS, lower segment cesarean section

Characteristics	Total number (*n*)	Percentage (%)
Mother age (mean)	26.33 ± 3.86	
Consanguinity		
Yes	139	9.9%
Obstetric Code		
Primiparous	498	35.6%
Multiparous	900	64.4%
Morbidity identified among mothers		
PIH (pregnancy-induced hypertension)		
Yes	211	15.1%
GDM (gestational diabetes mellitus)		
Yes	256	18.3%
Thyroid problem		
Yes	189	13.5%
Antenatal USG (ultrasound) findings		
Abnormal	117	8.4%
PROM (premature rupture of membranes)		
Yes	134	9.6%
Amniotic fluid status		
Normal	1238	88.6%
Oligohydramnios/Polyhydramnios	160	11.4%
Type of delivery		
LSCS	726	51.9%
Assisted vaginal	72	5.2%
Vaginal	600	42.9%

**Table 2:** Distribution of risk factors in the neonates. NICU, neonatal intensive care unit

Risk factors for hearing loss	Yes, *n *(%)	No, *n* (%)
Family history of hearing loss	1 (0.07%)	1,397 (99.9%)
NICU stay for >5 days	374 (26.8%)	1,024 (76.2%)
Hyperbilirubinemia requiring exchange transfusion or intense phototherapy	417 (21.8%)	981 (70.2%)
Asphyxia or hypoxic ischemic encephalopathy	86 (6.2%)	1,312 (93.8%)
Craniofacial malformations including microtia/atresia, ear dysplasia	1 (0.07%)	1,397 (99.9%)
No. of babies requiring mechanical ventilation	196 (14.0%)	1,202 (86.0%)

**Figure 1:**
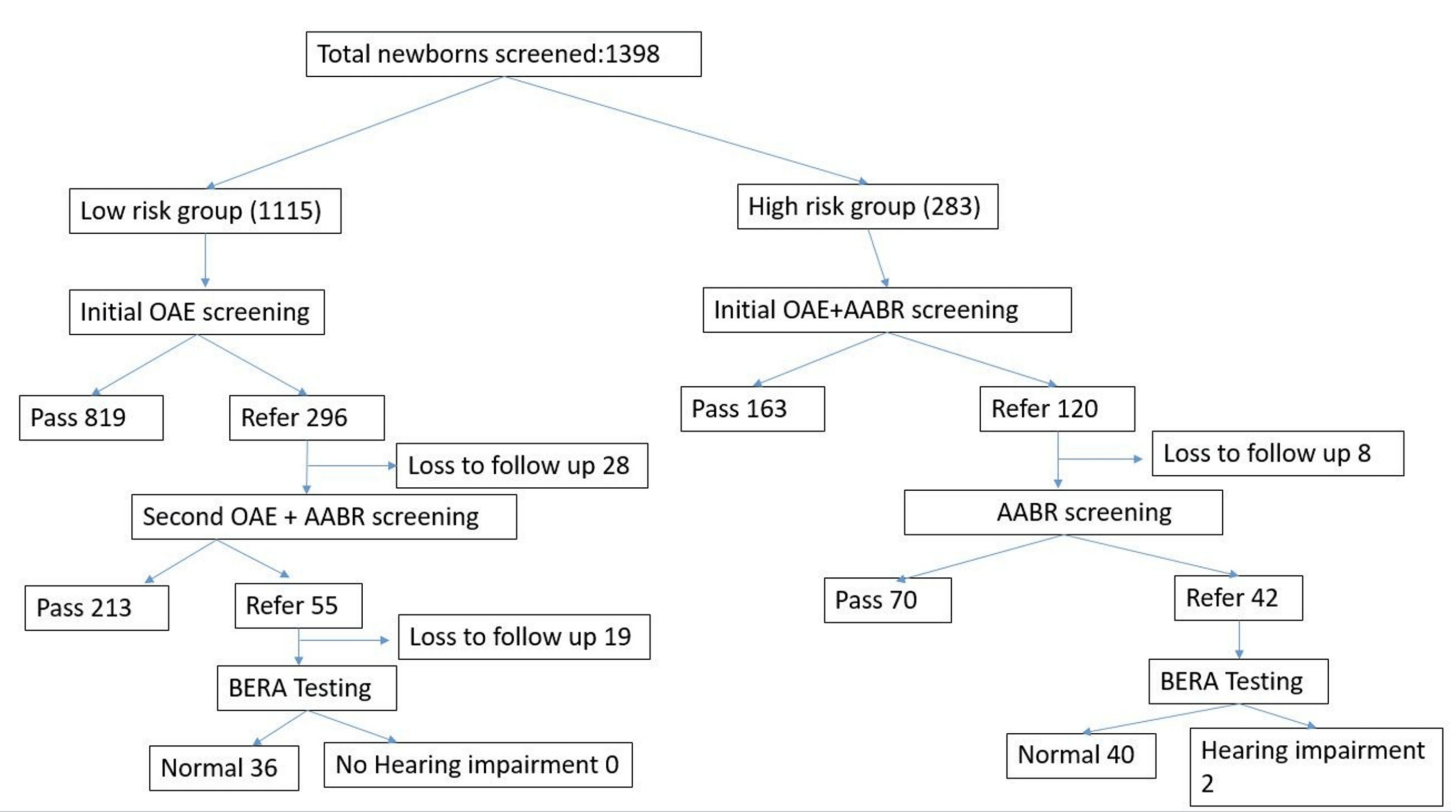
Flowchart of Screening and Diagnostic Protocol Image credit:  Anand Kumar G. OAE, otoacoustic emissions; AABR, automated auditory brainstem response; BERA, brainstem-evoked response audiometry

**Figure 2:**
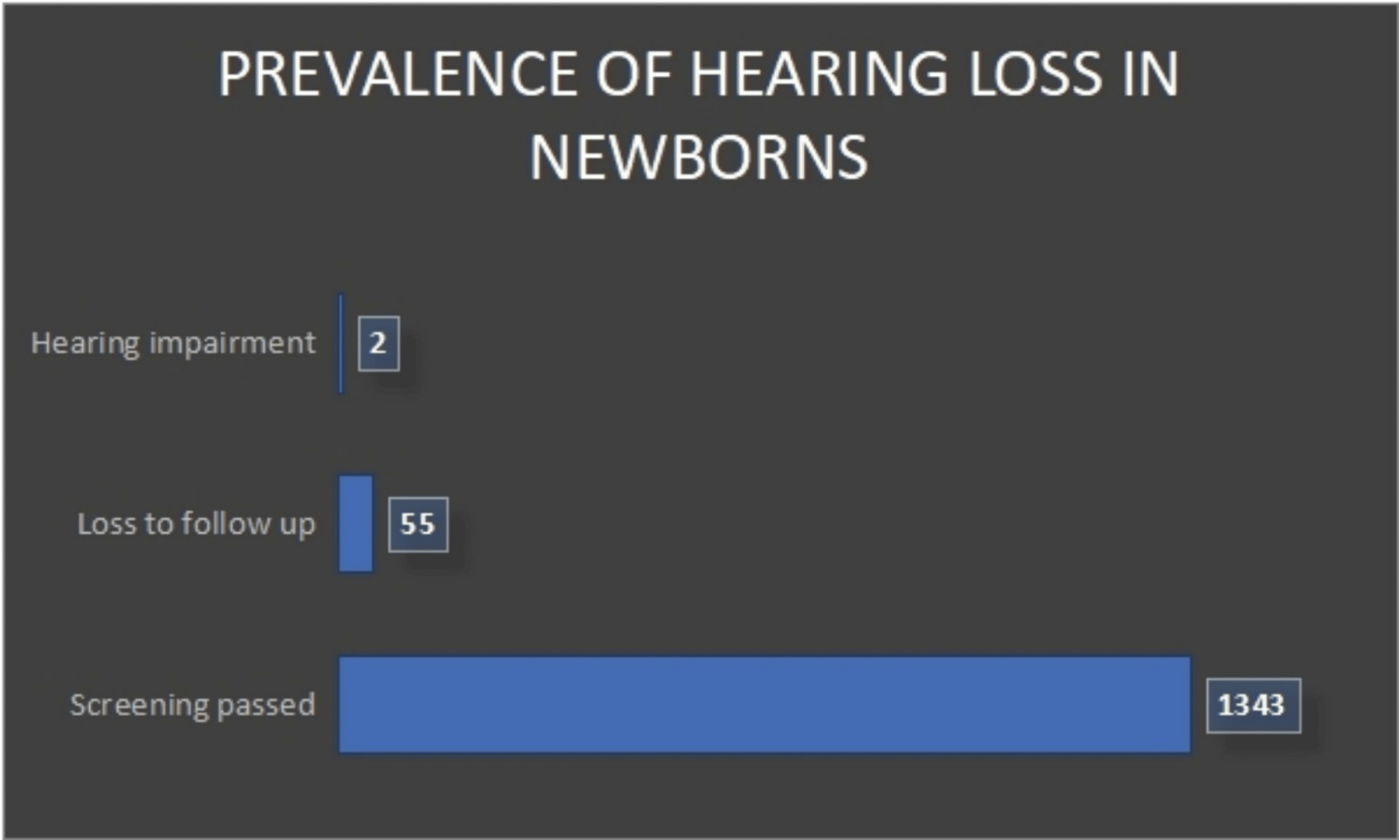
Prevalence of hearing loss.

In the initial OAEs screening, 1,115 low-risk neonates were evaluated, with 819 neonates passing and 296 neonates referred for additional testing (Figure 1). At the follow-up stage where both OAE and AABR were utilized, out of the 296 low-risk neonates who were initially referred, 213 passed, 55 were further referred, and 28 were lost to follow-up with none confirmed to have hearing loss after subsequent BERA testing. For the high-risk category, 283 newborns were initially screened with both OAE and AABR, resulting in 163 passing and 120 being referred. Upon follow-up testing with OAE and AABR, 70 neonates passed, 42 were referred for further evaluation, 8 did not return for follow-up, and 2 were ultimately diagnosed with hearing impairment after BERA testing. The screening was completed for 1,341 babies. 55 babies were lost to follow-up. Of these 2 babies (0.1%) had hearing loss (Figure 2, Table 3). 

**Table 3:** Initial and follow-up OAE screening results. OAE, otoacoustic emissions; AABR, automated auditory brainstem response; BERA, brainstem-evoked response audiometry

Risk category	Screening stage	Total screened	Passed	Referred	Lost to follow-up	Confirmed hearing loss after BERA
Low risk	Initial OAE	1115	819	296	-	-
	Follow-up OAE + ABR	296	213	55	28	0
High risk	Initial OAE + ABR	283	163	120	-	-
	Follow-up OAE + ABR	120	70	42	8	2

As noted in Table 4, in the low-risk category, out of 296 newborns who were referred for follow-up screening, a high compliance rate was observed, with 268 (90.5%) returning for additional testing. However, 28 did not return. Similarly, in the high-risk group, compliance was even higher, with 112 out of 120 referred newborns (93.3%) returning for their follow-up screening, while 8 did not return. 

**Table 4:** Compliance with follow-up screening.

Risk category	Total referred for follow-up	Number returning	Number not returning	Percentage returned
Low risk	296	268	28	90.5%
High risk	120	112	8	93.3%

For OAE, the sensitivity was reported at 66.7%, indicating that it correctly identified 66.7% of newborns with hearing loss (Table 5). The specificity of OAE was notably high at 98.8%, suggesting that it accurately identified 98.8% of newborns without hearing loss. However, the positive predictive value (PPV) was relatively low at 33.3%, meaning that only one-third of newborns identified by OAE as having a hearing issue have one, reflecting a significant rate of false positives. Conversely, the negative predictive value (NPV) was extremely high at 99.7%, indicating that nearly all newborns who passed the OAE test did not have hearing loss.

**Table 5:** Comparative sensitivity and specificity of OAE and BERA. OAE, otoacoustic emissions; BERA, brainstem-evoked response audiometry

Test type	Sensitivity (%)	Specificity (%)	Positive predictive value (%)	Negative predictive value (%)
OAE	66.7	98.8	33.3	99.7

## Discussion

The present study shows the critical importance of NHS in a tertiary care setting, with a focus on early identification and intervention for hearing impairments in newborns. This is crucial as undiagnosed hearing loss can profoundly affect a child's linguistic, social, and cognitive development. Our findings highlight the efficacy of utilizing both OAE and BERA in a sequential screening protocol to optimize the detection of auditory impairments in neonates. Two newborns were diagnosed with hearing loss. One baby had an external ear canal defect as right ear microtia. OAE of the left ear was referred. Bone conduction ABR was planned on follow-up. Ear reconstruction surgery is planned later. One more baby had severe profound hearing loss in both ears. Mother has been deaf and mute since childhood. Father was deaf since childhood. A cochlear implant in a hospital was planned. Both the babies with hearing loss were high-risk newborns. Both babies were diagnosed to have hearing loss within one to two months of life.

OAE has demonstrated high effectiveness as an initial screening tool, due to its non-invasive nature and rapid execution, which is particularly suited for the universal screening of newborns. The high specificity rate of 98.8% for OAE observed in our study aligns with other research, indicating its reliability in accurately identifying newborns without hearing loss. However, the sensitivity of OAE at 66.7% suggests that while effective, it may not detect all cases of hearing impairment, particularly auditory neuropathy and other neural hearing losses, which do not affect the cochlea directly. BERA, on the other hand, although more time-consuming and requiring a controlled environment, provides a comprehensive assessment of the auditory pathway, including the cochlea, auditory nerve, and brainstem. This makes BERA invaluable for confirming cases of hearing impairment detected through initial OAE screening, and for identifying cases that OAE may miss. BERA tests how the brainstem and brain react to sound. It is conducted in a controlled environment free from dust, sound, and electromagnetic interference. The forehead, vertex, and mastoid areas are cleaned and the infant wears earphones while surface electrodes are placed on the head and ears. To reduce myogenic activity, the neck is gently flexed. The test usually lasts for 30-45 minutes. The implementation of BERA in the second stage of screening is justified by its ability to detect both cochlear and neural dysfunctions, thus offering a safety net for neonates who pass the OAE but still have underlying hearing issues. A study by Shannon et al showed the sensitivity and specificity of BERA in detecting hearing loss are 100% and 86% respectively. The study's methodology, employing a two-step screening process where all neonates initially undergo OAE and those who refer proceed to BERA, ensures a robust mechanism for early detection. This combined approach not only leverages the strengths of both screening methods but also addresses their limitations, thereby enhancing overall screening accuracy. Our data reveal a significant detection of potential hearing impairments, which is critical for initiating timely interventions. The variation in NHS implementation across different healthcare settings noted in the literature suggests a need for standardized protocols, especially in tertiary care facilities that handle high-risk neonates [9]. The high compliance rates observed in our study for follow-up screenings indicate good parental engagement and institutional support, which are essential for the success of NHS programs.

A study revealed that children with hearing loss who were screened as newborns were diagnosed and received interventions (like hearing aids) significantly earlier by about 20-25 months than those who were not screened. This early intervention is crucial for better developmental outcomes in speech and communication skills [11]. Another study discussed the levels of evidence and the broad implementation of universal NHS (UNHS), indicating that early detection significantly reduces the age of intervention and improves developmental outcomes. It emphasized the importance of sensitive periods in auditory, speech, and language development [12]. In developing countries, challenges such as early discharge from birthing centers pose barriers to effective NHS. A study using AABR and TEOAE techniques found that screening within the first 48 hours post-birth led to significantly better outcomes, suggesting that early screening is feasible and crucial even in resource-limited settings [13]. A systematic review highlighted the lack of uniformity in NHS protocols globally and suggested that a combination of OAE and AABR screening provides the best balance of sensitivity and specificity. The review also addressed the need for further research on the most effective elements of screening programs to optimize outcomes [14]. Research also explored the experiences of parents whose children underwent NHS, noting the importance of communication style and the clarity of information provided during the screening process. Parents' perceptions and satisfaction with the screening process can significantly impact their engagement and compliance with follow-up interventions [15]. This study conducted a systematic review and meta-analysis of the effectiveness of UNHS programs. The findings emphasized that UNHS is effective in the early detection of permanent bilateral hearing loss in newborns, which is critical for timely intervention and support [16].

A comparison between UNHS and targeted, risk-based screening revealed that universal screening is more cost-effective. This study highlighted the balance between the costs of screening all newborns versus the higher medical costs and lost productivity associated with delayed diagnosis and treatment of hearing impairments [17]. A systematic review found that early identification followed by early intervention in children with hearing impairments is associated with better developmental outcomes. The review stressed the importance of high-quality evidence to support the effectiveness of NHS and subsequent interventions [18]. The study provided insights into the effectiveness of NHS across different settings, including neonatal intensive care units and well-baby nurseries. It found that the rate of newborns who failed hearing screening was consistent across different environments, underlining the need for universal screening irrespective of the newborn's health status at birth [19]. This study reviewed the first phase of a national population-based NHS program in England. It reported significant advantages in detecting bilateral permanent hearing loss early in life, supporting the effectiveness of UNHS in routine practice [20]. The results of this study have several implications for clinical practice and policy-making. First, the need for early and accurate detection of hearing impairments calls for widespread implementation of standardized NHS protocols, particularly in settings dealing with high-risk neonates. Second, the integration of OAE and BERA into routine neonatal care is emphasized.

The limitations of the study include a small sample size, loss to follow-up, and the fact that BERA was not performed on infants with abnormal BERA results at three to six months of age. Additionally, longitudinal follow-up of infants with hearing impairment was not conducted.

## Conclusions

Our study shows the significant role of NHS in detecting and managing hearing impairments in neonates, aligning with global research highlighting the importance of early intervention for optimal developmental outcomes. OAE and BERA have proven to be effective in detecting potential hearing loss. The high specificity of OAE makes it an excellent tool for initial screenings, efficiently ruling out hearing loss in most newborns and conserving medical resources for more targeted interventions. The study highlights the critical nature of early detection and timely interventions, which are pivotal in preventing the adverse impacts of undetected hearing loss on language, cognitive, and social development in children. Despite the success of the screening program, the study revealed challenges in ensuring follow-up compliance. Addressing these challenges is crucial for the success of NHS programs, as effective follow-up is necessary to confirm diagnoses and begin early interventions. The demographic analysis provided in the study helps in understanding the prevalence and distribution of hearing loss among different subgroups, which can aid in refining screening and intervention strategies. The findings advocate for the integration of systematic NHS protocols in all tertiary care settings, especially those serving high-risk populations.

Regular assessments and updates to these protocols can help in maintaining their effectiveness and efficiency. In conclusion, this study reinforces the necessity and effectiveness of comprehensive newborn hearing screening programs in tertiary care settings. It highlights the need for ongoing education, improved follow-up systems, and the universal adoption of NHS to ensure early detection and intervention for neonatal hearing loss, thereby supporting better developmental outcomes and quality of life for affected children.

## References

[1] Neumann K, Gross M, Bottcher P, Euler HA, Spormann-Lagodzinski M, Polzer M (2006). Effectiveness and efficiency of a universal newborn hearing screening in Germany. Folia Phoniatr Logop.

[2] Mehl AL, Thomson V (1998). Newborn hearing screening: the great omission. Pediatrics.

[3] Grill E, Hessel F, Siebert U, Schnell-Inderst P, Kunze S, Nickisch A, Wasem J (2005). Comparing the clinical effectiveness of different new-born hearing screening strategies. A decision analysis. BMC Public Health.

[4] Kalambe S, Gaurkar S, Jain S, Deshmukh P (2022). Comparison of otoacoustic emission (OAE) and brainstem evoked response audiometry (BERA) in high risk infants and children under 5 years of age for hearing assessment in western India: a modification in screening protocol. Indian J Otolaryngol Head Neck Surg.

[5] Soni A, Kanaujia SK, Kaushik S (2016). Brainstem evoked response audiometry (BERA) in neonates with hyperbillirubinemia. Indian J Otolaryngol Head Neck Surg.

[6] McClelland RJ, Watson DR, Lawless V, Houston HG, Adams D (1992). Reliability and effectiveness of screening for hearing loss in high risk neonates. BMJ.

[7] Uus K, Bamford J (2006). Effectiveness of population-based newborn hearing screening in England: ages of interventions and profile of cases. Pediatrics.

[8] Korver AM, Smith RJ, Van Camp G (2017). Congenital hearing loss. Nat Rev Dis Primers.

[9] Hrncic N, Goga A, Hrncic S, Hatibovic H, Hodzic D (2021). Factors affecting neonatal hearing screening follow-up in developing countries: one Insitution prospective pilot study. Medeni Med J.

[10] Lin HC, Shu MT, Lee KS, Ho GM, Fu TY, Bruna S, Lin G (2005). Comparison of hearing screening programs between one step with transient evoked otoacoustic emissions (TEOAE) and two steps with TEOAE and automated auditory brainstem response. Laryngoscope.

[11] Sininger YS, Grimes A, Christensen E (2010). Auditory development in early amplified children: factors influencing auditory-based communication outcomes in children with hearing loss. Ear Hear.

[12] Yoshinaga-Itano C (2004). Levels of evidence: universal newborn hearing screening (UNHS) and early hearing detection and intervention systems (EHDI). J Commun Disord.

[13] van Dyk M, Swanepoel de W, Hall JW 3rd (2015). Outcomes with OAE and AABR screening in the first 48 h--Implications for newborn hearing screening in developing countries. Int J Pediatr Otorhinolaryngol.

[14] Kanji A, Khoza-Shangase K, Moroe N (2018). Newborn hearing screening protocols and their outcomes: a systematic review. Int J Pediatr Otorhinolaryngol.

[15] Young A, Tattersall H (2007). Universal newborn hearing screening and early identification of deafness: parents' responses to knowing early and their expectations of child communication development. J Deaf Stud Deaf Educ.

[16] Edmond K, Chadha S, Hunnicutt C, Strobel N, Manchaiah V, Yoshinga-Itano C (2022). Effectiveness of universal newborn hearing screening: a systematic review and meta-analysis. J Glob Health.

[17] Kemper AR, Downs SM (2000). A cost-effectiveness analysis of newborn hearing screening strategies. Arch Pediatr Adolesc Med.

[18] Wolff R, Hommerich J, Riemsma R, Antes G, Lange S, Kleijnen J (2010). Hearing screening in newborns: systematic review of accuracy, effectiveness, and effects of interventions after screening. Arch Dis Child.

[19] Korres S, Balatsouras D, Papadimitriou A, Kandiloros D, Ferekidis E (2005). The prevalence of hearing loss in neonates admitted to a neonatal intensive care unit. Int J Pediatr Otorhinolaryngol.

[20] Korres SG, Balatsouras DG, Nikolopoulos T, Korres GS, Ferekidis E (2006). Making universal newborn hearing screening a success. Int J Pediatr Otorhinolaryngol.

